# Identification of the regulatory roles of water qualities on the spatio-temporal dynamics of microbiota communities in the water and fish guts in the Heilongjiang River

**DOI:** 10.3389/fmicb.2024.1435360

**Published:** 2024-08-21

**Authors:** Hongyu Jin, Lei Li, Wanqiao Lu, Zepeng Zhang, Yue Xing, Di Wu

**Affiliations:** ^1^Scientific Observing and Experimental Station of Fishery Resources and Environment in Heilongjiang River Basin, Ministry of Agriculture and Rural Affairs, Heilongjiang River Fishery Research Institute of Chinese Academy of Fishery Sciences, Harbin, China; ^2^National Agricultural Experimental Station for Fishery Resources and Environment in Fuyuan, Harbin, China

**Keywords:** Heilongjiang River, microbiota community, RDA, LEFSe, Pearson

## Abstract

The Heilongjiang River is one of the largest rivers in the cool temperate zone and has an abundant fish source. To date, the microbiota community in water samples and fish guts from the Heilongjiang River is still unclear. In the present study, water samples and fish guts were collected from four locations of the Heilongjiang River during both the dry season and the wet season to analyze the spatio-temporal dynamics of microbiota communities in the water environment and fish guts through 16s ribosome RNA sequencing. The water qualities showed seasonal changes in which the pH value, dissolved oxygen, and total dissolved solids were generally higher during the dry season, and the water temperature was higher during the wet season. RDA indicated that higher pH values, dissolved oxygen, and total dissolved solids promoted the formation of microbiota communities in the water samples of the dry season, while higher water temperature positively regulated the formation of microbiota communities in the water samples of the wet season. LEFSe identified five biomarkers with the most abundant difference at the genus level, of which *TM7a* was upregulated in the water samples of the dry season, and *SM1A02, Rheinheimera, Gemmatimonas*, and *Vogesella* were upregulated in the water samples of the wet season. Pearson analysis revealed that higher pH values and dissolved oxygen positively regulated the formation of *TM7a* and negatively regulated the formation of *SM1A02, Rheinheimera, Gemmatimonas*, and *Vogesella* (*p* < 0.05), while higher water temperature had the opposite regulatory roles in the formation of these biomarkers. The relative abundance of microbiota diversity in fish guts varies greatly between different fish species, even if the fishes were collected from the same water source, indicating that dietary habits and fish species may be key factors, affecting the formation and construction of microbiome community in fish gut. *P. glenii, P. lagowskii, G. cynocephalus*, and *L. waleckii* were the main fish resources, which were collected and identified from at least six sample points. RDA indicated that the microbiota in the water environment regulated the formation of microbiota community in the guts of *G. cynocephalus* and *L. waleckii* and had limited regulated effects on *P. glenii* and *P. lagowskii*. The present study identified the regulatory effects of water qualities on the formation of microbiota communities in the water samples and fish guts, providing valuable evidence for the protection of fish resources in the Heilongjiang River.

## 1 Introduction

The 16s ribosome RNA (16s rRNA) is present in all bacteria. 16S rRNA sequencing has been widely used to accurately identify the known bacteria and discover novel bacteria. 16s rRNA sequence analysis has been a standard method to detect the sequence differences in the hypervariable regions of the 16s rRNA gene, which plays essential roles in the taxonomy and identification of bacteria. An unknown bacteria can be identified by comparing the similarity of its 16s rRNA sequence with the known taxonomic bacteria in public databases, including Silva, which is a public database for the ribosomal RNA of wild organisms. 16S rRNA sequencing plays a particularly essential role in the bacterial identification of environments with mixed bacteria.

Microbiota plays a crucial role in a host organism due to its phylogenetic diversity and wild distribution. Studies on the microbiota in the fish gut have been a hot topic in recent years, which contributed to the analysis between microbes and host fish (Xiong et al., 2011; Ghanbari et al., 2015). The diversity of the microbiome community in the fish has been identified to be regulated by environmental factors, which play essential roles in the digestive and immune systems of host fish to adapt to the changes in environmental factors. The main microbiome in the fish included Protoctista, Fungi, Yeasts, Viruses, and members of the Bacteria and Archaea (Zhou et al., 2014), especially the bacteria, which has been identified as the dominant microbiota of the fish intestine (Ringø et al., 2012). The gastrointestinal tract can act as a reservoir of novel species, which has been a target tissue for the identification and discovery of bacteria. Thus, the majority of studies on the microbiota community were mainly focused on the gut or gastrointestinal ones of fish, which may affect the process of nutrition uptake, development, immunity, and resistance to pathogens (Tarnecki et al., 2017; Egerton et al., 2018; Wang et al., 2018). Certain other studies were focused on the microbiota community in the skin of fish because their microbiota acts as a defensive barrier against the infection of pathogens or pollutants (Merrifield and Rodiles, 2015).

Animal guts are hosts to a large number and wide variety of microbes, providing the animal hosts with more physiological functions (Kurilshikov et al., 2017; Ji et al., 2019). Previous studies have identified that aquatic animals select specific microbiota to exist in their gut tract, playing essential roles in the uptake of important nutrients to maintain the normal growth and development of the aquatic hosts, such as short-chain fatty acids, vitamins, and amino acids (Woznica et al., 2016; Fan and Li, 2019; Garibay-Valdez et al., 2020). Furthermore, increasing evidence indicates that the gut microbiota influences brain function and finally affects the response to stress (Ye et al., 2016). In fish, the gut microbiota has regulatory effects on the stress response by affecting the feeding behaviors and energy homeostasis of aquatic hosts (Mohanta et al., 2020; Cui et al., 2022).

The Heilongjiang River is one of the largest rivers in the cool temperate zone, with a total drainage area of about 1.84 million km^2^. The water content significantly changes throughout the year. The dry season is defined as the period when the surface water source in a watershed is depleted and mainly relies on groundwater to replenish water sources. It mainly happens in the season with little or even no rain. The wet season refers to the period when the water source mainly relies on rainfall for replenishment, which usually happens during the rainy season. The changes in water contents from the dry season to the rainy season may lead to compositional changes in the microbiota composition and diversity of the water environment (Smith A. P. et al., 2015; Zhang et al., 2015). Environmental changes could alter the biomass and diversity of ecological communities, thus affecting the functionality and multifunctionality of an ecosystem (Soliveres et al., 2016).

In the present study, the water qualities were measured in the four locations of the Heilongjiang River and the Huma River during both the wet season and dry season. In addition, the spatio-temporal dynamics of microbiota communities in the water environment and the microbiota communities in the fish gut were also investigated based on 16s rRNA sequencing in order to carry out the distribution, diversity, and abundance of microbiota. The present study provided valuable evidence to carry out the regulatory effects of water qualities on the formation of microbiota communities in water environments and the fish gut.

## 2 Materials and methods

### 2.1 Measurement of the water qualities in the wet season and the dry season

**Figure 1:**
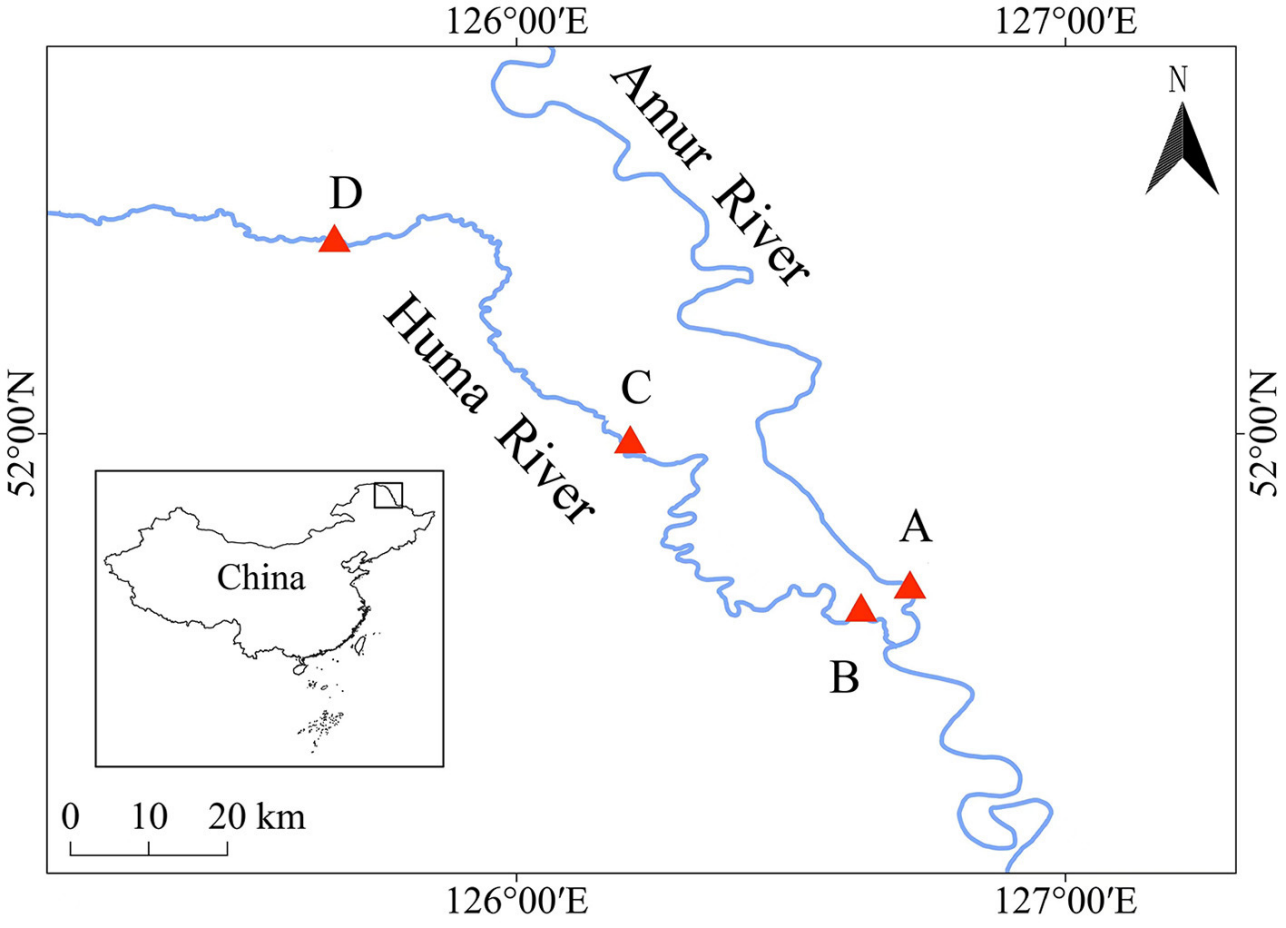
Locations to collect water samples and fish samples during the dry season and the wet season. A, B, C, and D indicate the locations to collect water samples and fish samples during the dry season and the wet season.

Water samples and fish samples were collected from the mainstream of the Heilongjiang River (location A, 126°43′7″, 51°43′6″) and the upper (location B, 126°37′40″, 51°40′44″), middle (location C, 126°34′28″, 51°40′2″), and lower (location D, 125°40′8″, 52°21′9″) reaches of the Huma River, which is a tributary of the Heilongjiang River (Figure 1). The water samples and fish samples were collected from these four locations in both the wet season (August) and the dry season (May). The water samples were collected from the water square, with an area of 1 km^2^ for each location. A total of 2 L of water samples were obtained from the center and four corners of this square with a 0.5 m depth from the surface of the water body, and the sampling time was 9 a.m. The water samples were mixed together to measure the water qualities and filter the microbiota community from each water environment. The water qualities in each water environment were measured by using a multifunctional water quality detector (Henxin 86031, China), including pH value, water temperature, dissolved oxygen, saline concentration, and total dissolved solids.

### 2.2 Isolation of microbiota from water samples and fish samples

A total of 1 L of mixed water sample from each location was collected in sterile jars, and a manual pump with a sterile filtration unit was immediately used to filter the water on 0.22-μm sterile membranes in triplicate. Filters were immediately frozen in liquid nitrogen and stayed at −80°C until DNA extraction.

A total of eight sampling sites were designed with a 1,000-m river section for each sampling location. Five mesh gillnets (mesh: 1 cm, 2 cm, 4 cm, 6 cm, and 8 cm; net height: 1 m; and net length: 200 m) and three ground cages (80 m long, 0.4 m high, and mesh 0.5 cm) were prepared to collect fish samples from each sampling location. Mesh gillnets were used to capture pelagic fish in the middle and upper layers, while ground cages were used to capture benthic fish. Fish samples collected during the wet season and the dry season are listed in Supplementary Tables 1, 2, including the total length, body length, and body weights of each fish sample. Each fish species at each location was collected from nine individuals. The whole gut was collected from each individual for every fish species. Three guts from each fish species were pooled together to form a biological replicate, and three biological replicates were prepared. All of the gut samples were immediately frozen in liquid nitrogen and stayed at −80°C until DNA extraction.

### 2.3 DNA extraction, PCR amplification, and sequencing

Filters were washed with three washing solutions (Tris-HCl, EDTA, and Triton X-100) prior to the DNA extraction in order to eliminate the effects of extracellular DNA and enhance the possibility of PCR amplification (Fortin et al., 2004). The total microbial genomic DNA from water samples and fish gut samples was extracted by using the DNeasy PowerSoil Pro Kit (QIAGEN, U.S.), according to the manufacturer's instructions. The DNA quality was determined by 1.0% agarose gel electrophoresis, and the concentration of DNA was measured by the NanoDrop ND-2000 spectrophotometer (Thermo Scientific Inc., USA). The highly variable region V3–V4 of the bacterial 16S rRNA gene was amplified with the primers 338F (5'-ACTCCTACGGGGGGCAG-3') and 806R (5'-GACTACHVGGGTWTCTAAT-3') through Eppendorf AG (Eppendorf, Germany) (Xu et al., 2016a,b). The PCR amplification was performed with a 20-reaction mixture, including 4 μl of 5 × Fast Pfu buffer, 2 μl of 2.5-mM dNTPs, 0.8 μl of each primer (5 μM), 0.4 μl of Fast Pfu polymerase, 0.2 μl of BSA, 10 ng of template DNA, and ddH2O. The thermal amplification cycle conditions were as follows: initial denaturation at 95°C for 3 min, 30 cycles of denaturation at 95°C for 30 s, annealing at 55 °C for 30 s, extension at 72°C for 45 s, final extension at 72°C for 10 min, and termination at 4°C. All samples were repeated three times. The PCR products were determined by using the 2% agarose gel and purified using the AxyPrep DNA Gel Extraction Kit (Axygen Biosciences, Union City, CA, USA), according to the manufacturer's instructions. The purified PCR products were quantified using a Quantus™ Fluorometer (Promega, USA).

The purified PCR products were then used to construct the library by using the NEXTFLEX Rapid DNA-Seq Kit, according to the manufacturer's instruments. Illumina's Miseq PE300 was used to perform the amplicon sequencing (Shanghai Meiji Biomedical Technology Co., Ltd.).

### 2.4 Data and statistical analysis

Raw FASTQ files for all water samples and fish gut samples were de-multiplexed using an in-house perl script, quality-filtered by fastp version 0.19.6 (Chen et al., 2018), and merged using FLASH version 1.2.7 (Magoč and Salzberg, 2011). Then, the optimized sequences were clustered into operational taxonomic units (OTUs) using UPARSE 7.1 with a 97% sequence similarity level (Edgar, 2013). The most abundant sequence for each OTU was selected as a representative sequence. The taxonomy of each OTU representative sequence was analyzed by RDP Classifier version 2.2 (Lan et al., 2012) against the 16S rRNA gene database (Silva v138) using a confidence threshold of 0.7 to obtain the annotated taxonomy table (Quast et al., 2012).

To explore whether there are differences in the composition of microbial communities in different water samples, principal component analysis (PCA) was performed using an R-mode PCA in R software (Ihaka and Gentleman, 1996), based on OTUs. R package vegan (Oksanen et al., 2013) was used to reveal the relationship between water qualities and environmental microbiota community through redundancy analysis (RDA). Linear discriminant analysis effect size (LEFSe) was performed to discover environmental biomarkers with statistical differences in the water samples between the dry season and the wet season, using R package microeco (Liu et al., 2021a). The relationship between water qualities and biomarkers was performed by using the Pearson correlation analysis. The vegan package (Oksanen et al., 2013)-based RDA was also used to demonstrate the effects of environmental biomarkers on the formation of intestinal microbiota community in the same fish species from different sample points, according to the OTU data of different fishes and the abundance of the environmental biomarkers. All data visualizations were based on the R package ggplot2 (Wilkinson, 2011).

### 2.5 Statistical analysis

SPSS Statistics 23.0 (IBM, Armonk, NY, USA) was used to conduct all statistical analyses. Statistical differences in growth traits of the same fish species were identified by an independent *t*-test and a one-way analysis of variance, followed by the least significant difference. Quantitative data were expressed as mean ± standard deviation. A value of *p* < 0.05 was considered to be statistically significant.

## 3 Results

### 3.1 The changes in water qualities between the dry season and the wet season

The water qualities were measured in the water samples, collected during the dry season and the wet season, including the pH value, water temperature, dissolved oxygen, saline concentration, and total dissolved solids. The water qualities showed a significant difference between the wet season and the dry season. According to Tables 1, 2, the pH values, dissolved oxygen, and total dissolved solids of the four locations during the dry season were generally higher than those of the wet season, while the water temperatures during the wet season were significantly higher than those of the dry season. The pH values of the four locations during the dry season ranged from 8.72 to 8.89, while they ranged from 6.5 to 6.85 during the wet season. The dissolved oxygen during the dry season ranged from 7.6 to 8.1 mg/L, while it ranged from 6.5 to 6.9 mg/L during the wet season. The water temperatures during the wet season were higher than 22°C, while they were lower than 18°C during the dry season. However, the changes in water contents between the wet season and the dry season did not result in significant changes in saline concentrations.

**Table 1:** Water qualities of four locations during the dry season.

	**pH**	**T (°C)**	**DO (mg/L)**	**S (‰)**	**TDS**
a	8.89	13.7	8.1	0.05	54.1
b	8.72	18.2	7.6	0.03	44.2
c	8.73	16.1	7.8	0.03	41.3
d	8.68	15.3	7.7	0.02	41

T, temperature; DO, dissolved oxygen; S, saline concentration; TDS, total dissolved solids.

**Table 2:** Water qualities of four locations during the wet season.

	**pH**	**T (°C)**	**DO (mg/L)**	**S (‰)**	**TDS**
a	6.78	28.5	6.8	0.07	38
b	6.5	22	6.5	0.04	35
c	6.85	25.1	6.9	0.01	34
d	6.8	26.2	6.8	0.03	33

T, temperature; DO, dissolved oxygen; S, saline concentration; TDS, total dissolved solids.

### 3.2 The changes in bacterial phylum in water samples between the wet season and the dry season

**Figure 2:**
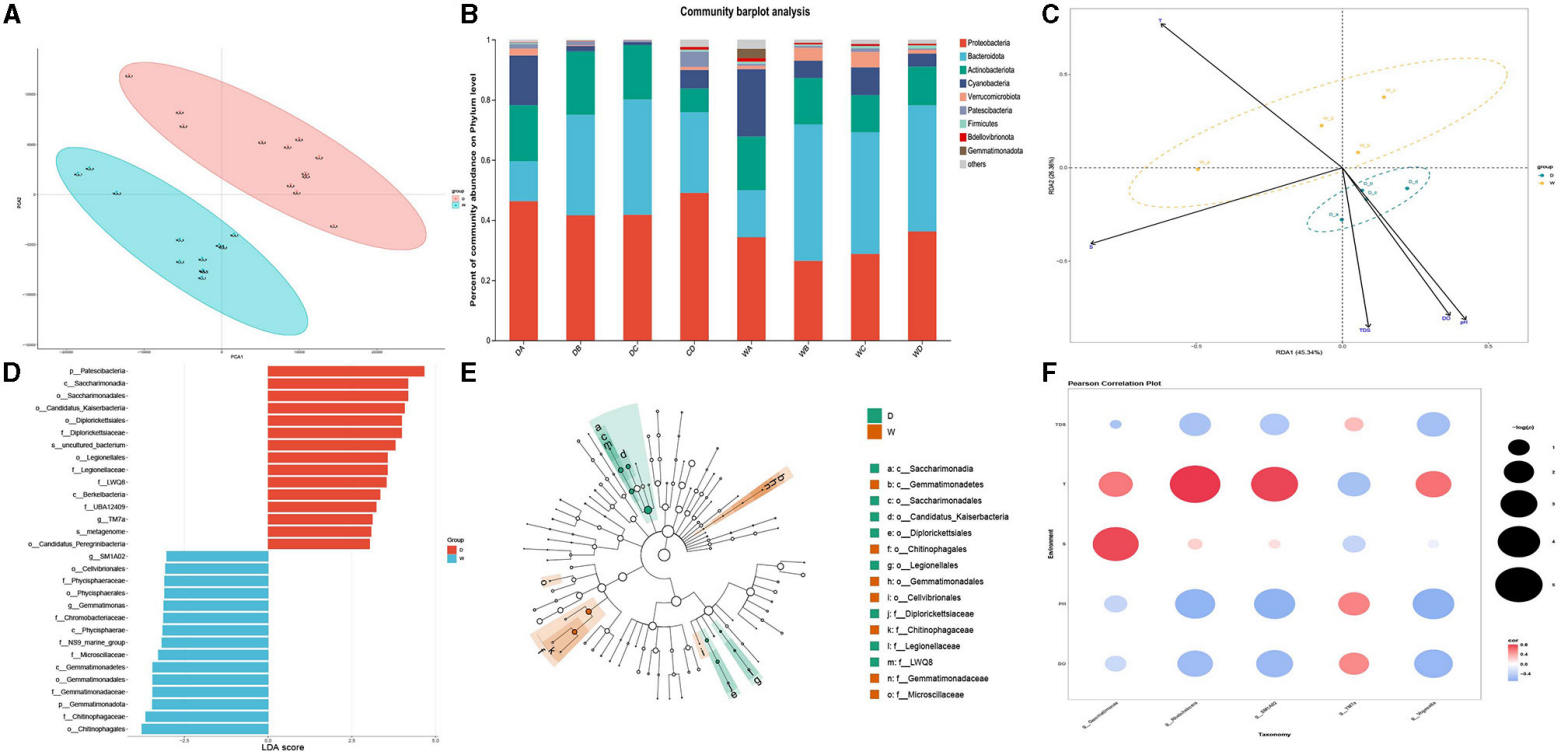
Comparison of microbiota community in individual water samples. D indicates the water samples collected from the dry season. W indicates the water samples collected from the wet season. a, b, c, and d indicate the four locations to collect water samples. **(A)** Indicates the principal component analysis for the microbiota community in the water samples between the dry season and wet season. **(B)** Indicates the phylum distribution as a percentage of the total number of identified sequences in an individual water sample. **(C)** Indicates the RDA analysis for the effects of water qualities on the composition of the microbiota community in water samples. **(D)** Indicates the top 15 biomarkers with the greatest difference in the water between the dry season and wet season, analyzed by the LEFSe. **(E)** Indicates the cladogram of LEFSe. **(F)** Indicates the correlation between water qualities and the diversity of biomarkers. Colors indicate the r^2^, and plot size indicates the significance.

The PCA of water samples was conducted by the three biological replicates collected during the wet season and dry season (Figure 2A). Clear separations were observed between the water samples collected during the wet season and the dry season, indicating that the present study constructed a strongly reliable model with a low risk of overfitting to identify the microbiome community in the water samples collected during the wet season and the dry season.

Although the water samples in the present study had a similar bacterial taxonomic composition, the relative abundance at the bacterial phyla level showed a significant difference. The changes in water qualities resulted in a significant difference in the microbiota in water samples between the wet season and the dry season. The dominant bacterial phylum in the water samples of the four locations during the dry season was Proteobacteria, followed by Bacteroidota and Actinobacteriota, while the most abundant bacterial phyla in the water samples during the wet season were Bacteroidota, except location A. Proteobacteria was the dominant bacterial phylum in the water sample of location A, followed by Bacteroidota and Cyanobacteria. However, the abundance of Proteobacteria significantly decreased during the wet season compared to that during the dry season. The relative abundances for each water sample are shown in Figure 2B.

RDA was used to identify the effects of different water qualities on the formation of microbiome communities between different water samples collected during the wet season and the dry season (Figure 2C). The RDA indicated that higher pH values, dissolved oxygen, and TDS had positively regulated effects on the formation of the microbiome community in the water samples during the dry season, while higher water temperature had positively regulated effects on the formation of the microbiome community in the water samples during the wet season. However, saline concentration had limited effects on the regulation of the microbiome community between two different seasons.

LEFSe was used to identify the biomarkers and representative microorganisms in the water samples between the wet season and the dry season (Figures 2D, E). The present study identified the 15 biomarkers with the greatest differences between the wet season and the dry season. The most differential biomarkers were Patescibacteria from phylum level, Saccharimonadia from class level, and Saccharimonadales from order level in the water samples from the dry season, while the most differential biomarkers were Chitinophagates from order level, Chitinophagaceae from family level, and Gemmatimonadota from phylum level in the water samples from the wet season. Five biomarkers showed a significant difference at the bacterial genus level in the water samples between the dry season and the wet season, of which *TM7a* was upregulated in the water samples of the dry season, and *SM1A02, Rheinheimera, Gemmatimonas*, and *Vogesella* were upregulated in the water samples of the wet season.

Pearson analysis identified the effects of water qualities on the formation of five biomarkers, which showed the most significant difference in the water samples between the dry season and the wet season. Figure 2F reveals that higher pH values and dissolved oxygen positively regulated the formation of *TM7a* and negatively regulated the formation of *SM1A02, Rheinheimera, Gemmatimonas*, and *Vogesella* (*p* < 0.05). However, the higher temperature had negative effects on the formation of *TM7a* and had positive effects on the formation of *SM1A02, Rheinheimera, Gemmatimonas*, and *Vogesella* (*p* < 0.05), which was consistent with the results of RDA.

### 3.3 Identification of the bacterial phylum in fish guts during the wet season and the dry season

**Figure 3:**
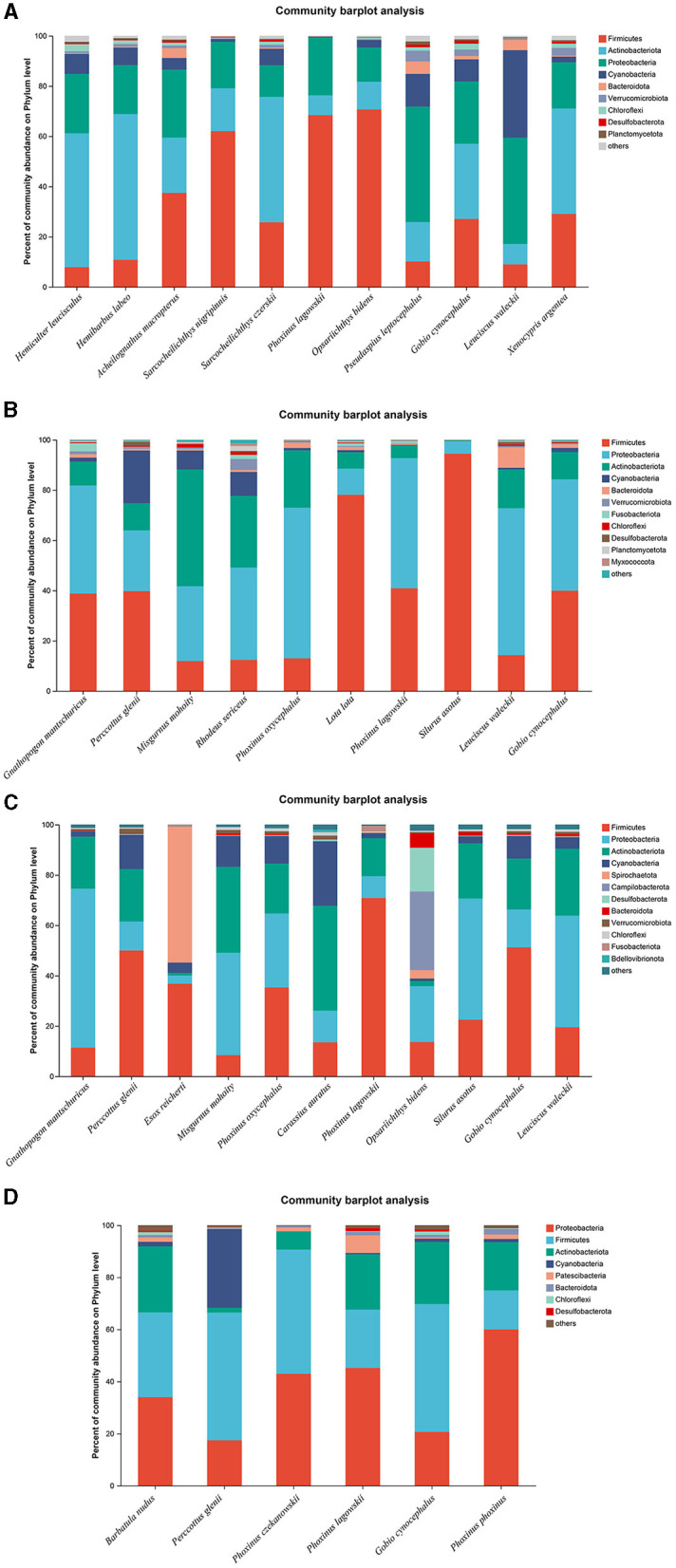
Phylum distribution as a percentage of the total number of identified sequences in fish guts collected during the dry season. **(A–D)** Indicate location **(A)**, location **(B)**, location **(C)**, and location **(D)**, respectively.

The microbiota in the guts of different fish species showed significant differences at the bacterial phyla level, collected from the same location during the dry season and the wet season. A total of 11, 10, 11, and 6 fish species were collected and identified from locations A, B, C, and D during the dry season, respectively. In location A, Firmicutes was the dominant bacterial phylum in *Sarcocheilichthys nigripinnis, Phoxinus lagowskii, Opsariichthys bidens*, and *Acheilognathus macropterus*. The most abundant bacterial phylum in *Hemiculter leucisculus, Hemibarbus labeo, Sarcocheilichthys czerskii*, and *Xenocypris argentea* was Actinobacteriota. Proteobacteria was the most abundant bacterial phylum in *Leuciscus waleckii* and *Pseudaspius leptocephalus*. However, the abundances of Firmicutes, Actinobacteriota, and Proteobacteria were almost the same in *Gobio cynocephalus* (Figure 3A). In location B, Firmicutes was the dominant bacterial phylum in *Silurus asotus, Lota lota*, and *Perccottus glenii*. The most abundant bacterial phylum in *L. waleckii, Phoxinus oxycephalus*, and *Rhodeus sericeus* was Proteobacteria. The abundances of Firmicutes and Proteobacteria in *Gnathopogon mantschuricus, P. lagowskii*, and *G. cynocephalus* were almost the same, which were dramatically higher than those of the other bacterial phyla. However, Actinobacteriota was the most abundant bacterial phylum in *Misgurnus mohoity* (Figure 3B). In location C, Firmicutes was the dominant bacterial phylum in *P. lagowskii, G. cynocephalus*, and *P. glenii*, while Proteobacteria was the most abundant bacterial phylum in *L. waleckii, G. mantschuricus*, and *S. asotus*. The most abundant bacterial phyla in *Esox reicherti, Carassius auratus*, and *O. bidens* were Spirochaetota, Actinobacteriota, and Campilobacterota, respectively (Figure 3C). In location D, Proteobacteria was the most abundant bacterial phylum in *Phoxinus phoxinus* and *P. lagowskii*, while Firmicutes was the dominant bacterial phylum in *G. cynocephalus* and *P. glenii*. The abundances of Firmicutes and Proteobacteria showed no difference in *Barbatula nudus* and *Phoxinus czekanowskii* (Figure 3D). The relative abundances for the guts of each fish sample are shown in Figure 3, collected during the dry season.

**Figure 4:**
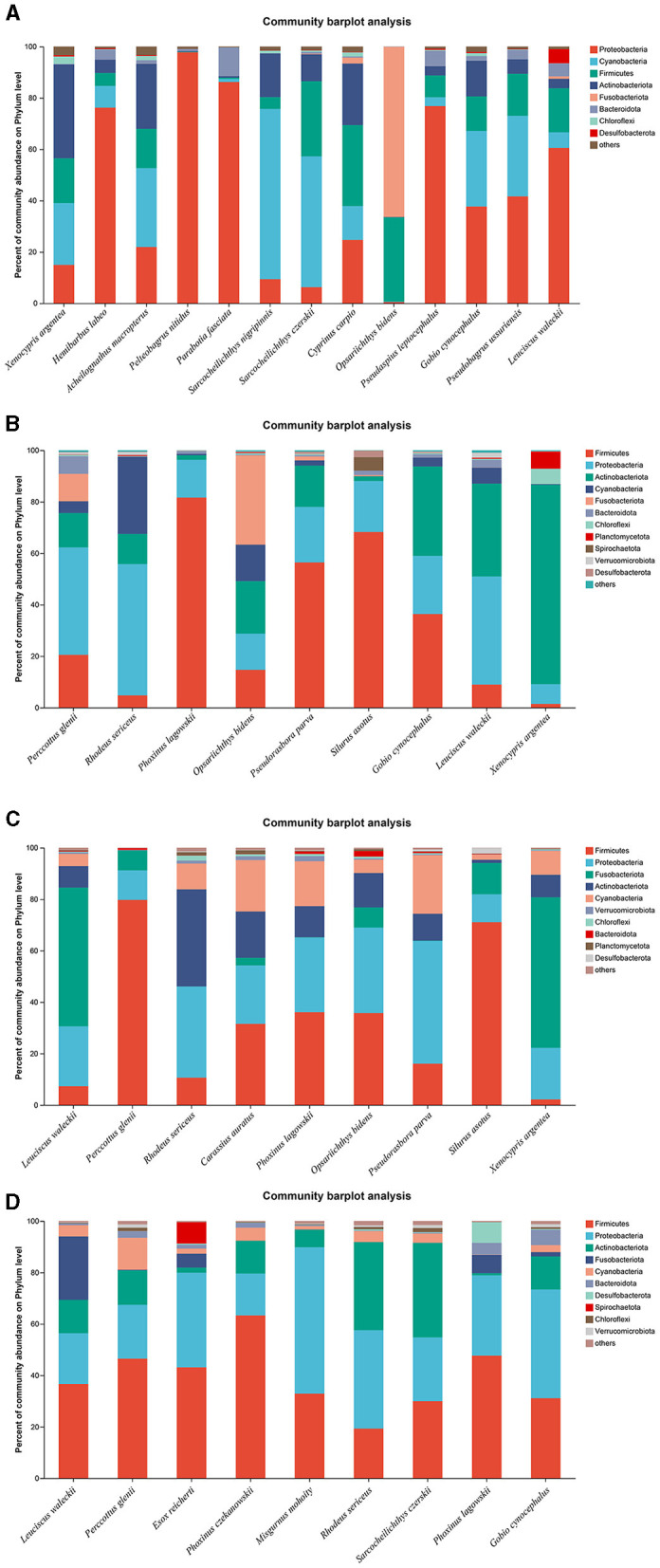
Phylum distribution as a percentage of the total number of identified sequences in fish guts collected during the wet season. **(A–D)** Indicate location **(A)**, location **(B)**, location **(C)**, and location **(D)**, respectively.

A total of 13, 9, 9, and 9 fish species were collected and identified from locations A, B, C, and D during the wet season, respectively. In location A, Proteobacteria was the dominant bacterial phylum in *H. labeo, Pelteobagrus nitidus, Parabotia fasciata, P. leptocephalus*, and *L. waleckii*, while Cyanobacteria was the most abundant bacterial phylum in *S. nigripinnis* and *S. czerskii*. The most abundant bacterial phyla in *O. bidens, X. argentea*, and *Cyprinus carpio* were Fusobacteriota, Actinobacteriota, and Firmicutes, respectively. However, the dominant bacterial phyla in *G. cynocephalus* and *Pseudobagrus ussuriensis* were Proteobacteria and Cyanobacteria, respectively, which showed no significant difference (Figure 4A). In location B, Firmicutes was the dominant bacterial phylum in *P. lagowskii, S. asotus*, and *Pseudorasbora parva*, while the most abundant bacterial phylum in *P. glenii* and *R. sericeus* was Proteobacteria. The dominant bacterial phyla in *G. cynocephalus* were Actinobacteriota, Firmicutes, and Proteobacteria, while the abundant bacterial phyla in *L. waleckii* were Actinobacteriota and Proteobacteria. The most abundant bacterial phylum in *X. argentea* was Actinobacteriota (Figure 4B). In location C, Fusobacteriota was the most abundant bacterial phylum in *L. waleckii* and *X. argentea*, while the dominant bacterial phylum in *S. asotus* and *P. glenii* was Firmicutes. Firmicutes and Proteobacteria were the abundant bacterial phyla in *P. lagowskii* and *O. bidens*, and the main bacterial phyla in *R. sericeus* were Proteobacteria and Actinobacteriota. Proteobacteria was the most abundant bacterial phylum in *P. parva* (Figure 4C). In location D, Firmicutes and Proteobacteria were the most abundant bacterial phyla in *P. czekanowskii* and *M. mohoity*, respectively. Firmicutes and Proteobacteria were the dominant bacterial phyla in *G. cynocephalus, P. lagowskii, E. reicherti*, and *P. glenii*, while the abundant bacterial phyla in *R. sericeus* and *S. czerskii* were Firmicutes, Proteobacteria, and Actinobacteriota (Figure 4D). The relative abundances for the guts of each fish sample are shown in Figure 4, collected during the wet season.

### 3.4 The regulatory effects of microbiota communities between water samples and fish guts

**Figure 5:**
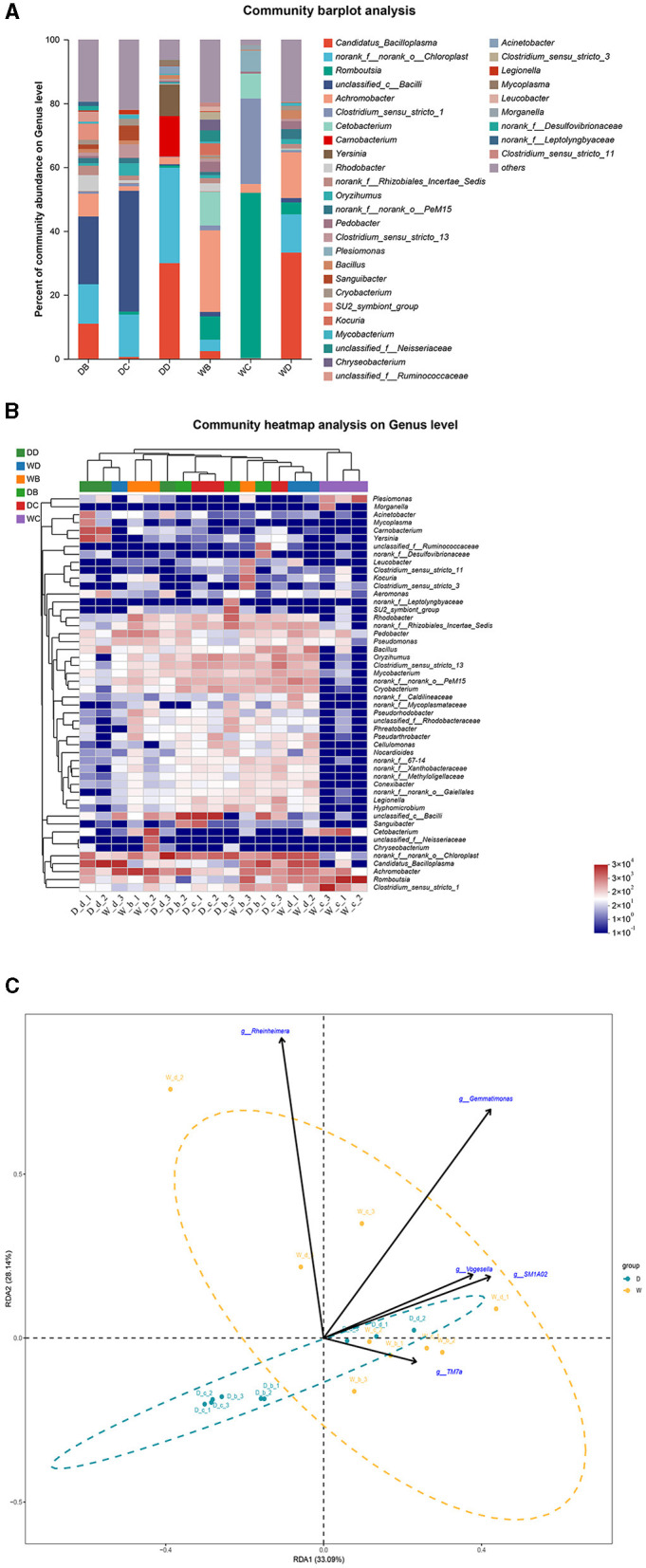
A comparison of microbiota community in the guts of *Perccottus glenii* collected from different sample points. D indicates the water samples collected from the dry season. W indicates the water samples collected from the wet season. b, c, and d indicate the different locations to collect fish samples. **(A)** Indicates genus distribution as a percentage of the total number of identified sequences in the guts of *P. glenii* collected from different sample points. **(B)** Indicates the clustering diagram of bacteria at the genus level. The horizontal coordinates represent the sample names and the clustering results of the samples, while the vertical coordinates represent the bacteria and the clustering results of the bacteria. The colors represent the abundance of bacteria in the samples. **(C)** Indicates the RDA analysis for the effects of five biomarkers on the composition of the microbiota community in the guts of *P. glenii*.

The fish samples were collected from a total of eight sample points (four sample points during the dry season and four sample points during the wet season) in the present study. *P. glenii, P. lagowskii, G. cynocephalus*, and *L. waleckii* were the main fish resources, which were collected and identified from at least six sample points out of these eight sample points. The microbiota community showed a significant difference at the bacterial genera level in the fish guts of the same fish species, collected from different locations. The microbiota changes at the bacterial genera level showed the location specificity in the same fish species. In the present study, *P. glenii* was collected from six locations. *Candidatus_Bacilloplasma, norank_f__norank_o__Chloroplast*, and *unclassified_c__Bacilli* were the most abundant bacterial genera in the guts of *P. glenii* from location B during the dry season, while *Candidatus_Bacilloplasma* and *unclassified_c__Bacilli* were the dominant bacterial genera in the guts from location D during the dry season. *Unclassified_c__Bacilli, Achromobacter, Romboutsia*, and *Candidatus_Bacilloplasma* were the most abundant bacterial genera in the guts from location C during the dry season and from locations B, C, and D during the wet season, respectively; of which, the abundances were dramatically higher than the other bacterial genera (Figures 5A, B). RDA revealed that the composition of the microbiota community in the guts of *P. glenii* did not fully separate between the two seasons. Thus, the biomarkers have limited effects on the formation of microbiota communities in the guts of this species (Figure 5C).

**Figure 6:**
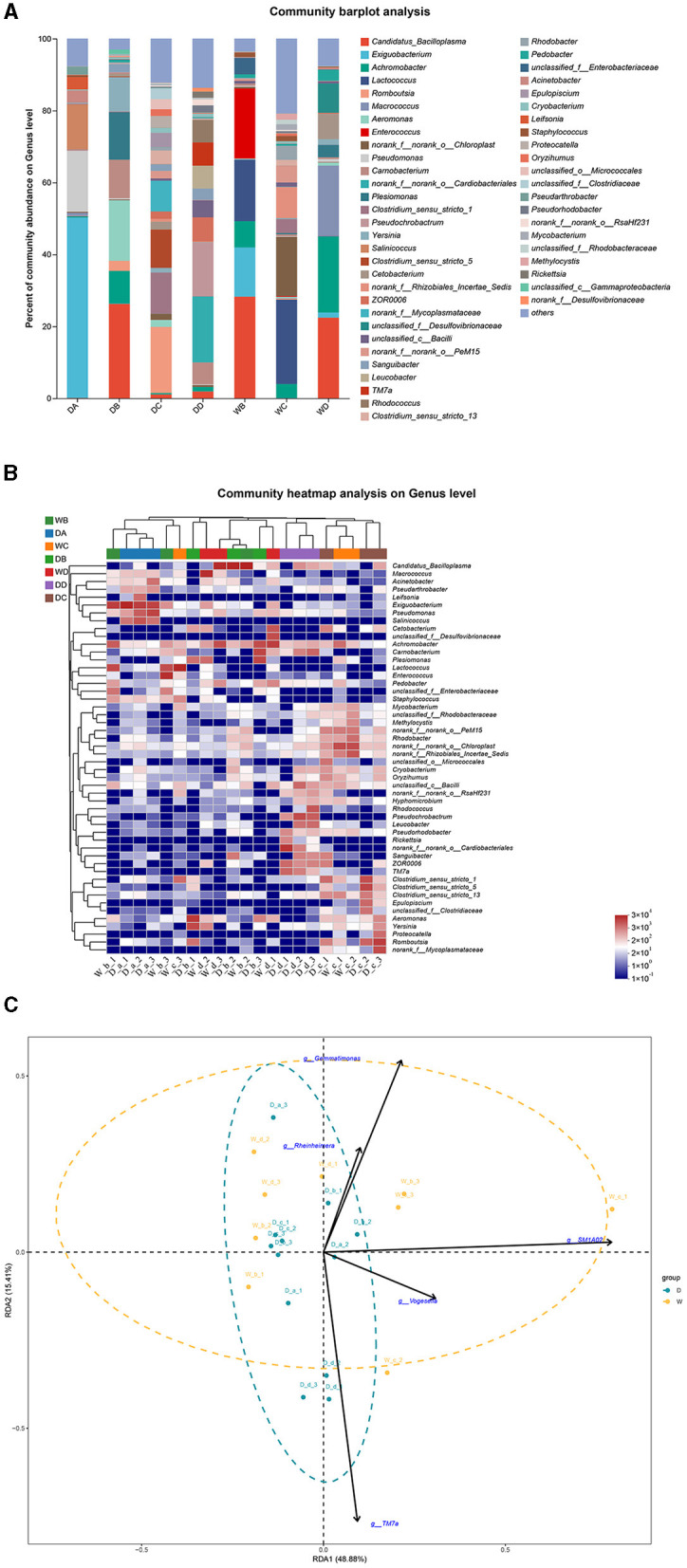
A comparison of microbiota community in the guts of *Phoxinus lagowskii* collected from different sample points. D indicates the water samples collected from the dry season. W indicates the water samples collected from the wet season. a, b, c, and d indicate the different locations to collect fish samples. **(A)** Indicates genus distribution as a percentage of the total number of identified sequences in the guts of *P. lagowskii* collected from different sample points. **(B)** Indicates the clustering diagram of bacteria at the genus level. The horizontal coordinates represent the sample names and the clustering results of the samples, while the vertical coordinates represent the bacteria and the clustering results of the bacteria. The colors represent the abundance of bacteria in the samples. **(C)** Indicates the RDA analysis for the effects of five biomarkers on the composition of the microbiota community in the guts of *P. lagowskii*.

In the present study, *P. lagowskii, G. cynocephalus*, and *L. waleckii* were collected from seven locations. *Exiguobacterium, Romboutsia, norank_f__norank_o__Cardiobacteriales*, and *Lactococcus* were the most abundant bacterial genera in the guts of *P. lagowskii* from locations A, C, and D during the dry season and from location C during the wet season, respectively. *Candidatus_Bacilloplasma* was the dominant bacterial genus in the fish guts from location B during the dry season and from locations B and D during the wet season (Figures 6A, B). RDA revealed that no clear separation was observed in the composition of the microbiota community in the guts of *P. lagowskii* between the two seasons. Thus, the biomarkers also have limited effects on the formation of microbiota communities in the guts of this species (Figure 6C).

**Figure 7:**
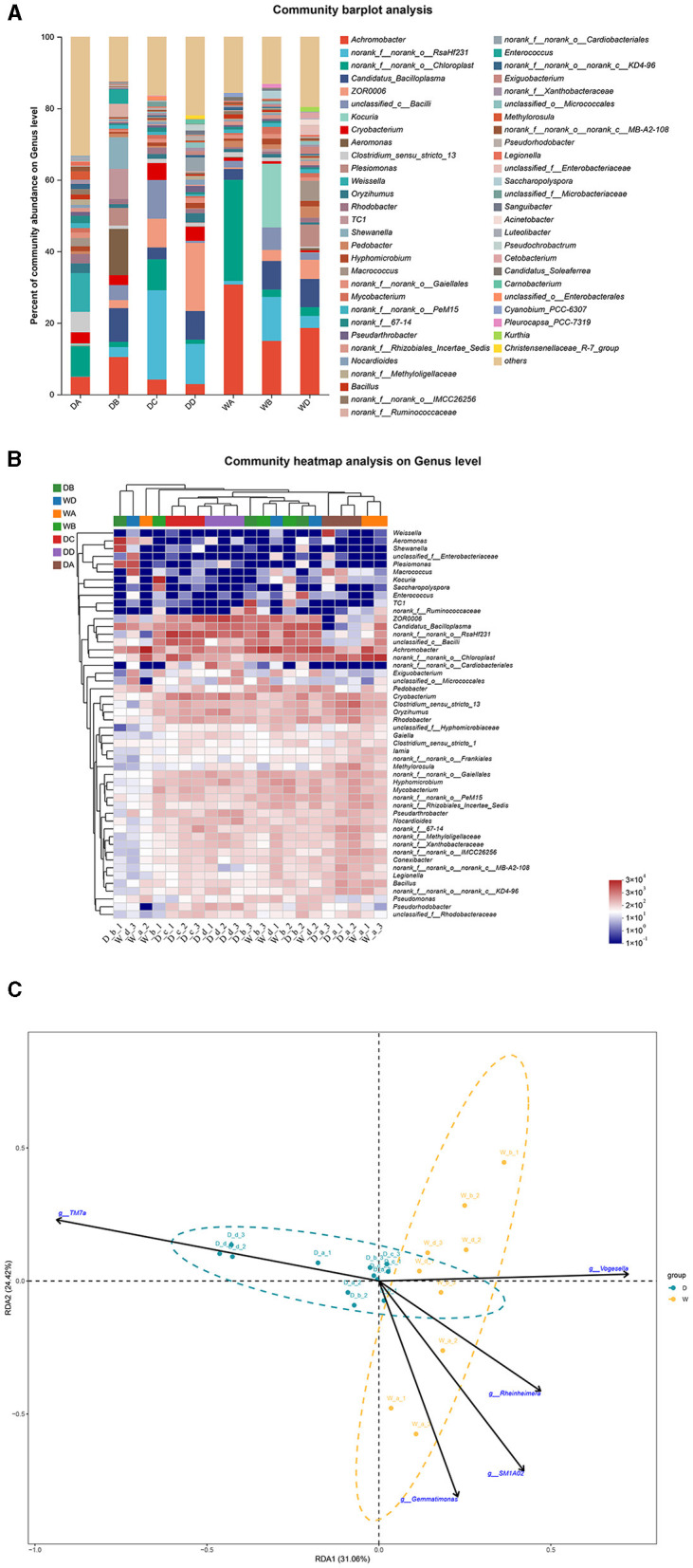
A comparison of microbiota community in the guts of *Gobio cynocephalus* collected from different sample points. D indicates the water samples collected from the dry season. W indicates the water samples collected from the wet season. a, b, c, and d indicate the different locations to collect fish samples. **(A)** Indicates genus distribution as a percentage of the total number of identified sequences in the guts of *G. cynocephalus* collected from different sample points. **(B)** Indicates the clustering diagram of bacteria at the genus level. The horizontal coordinates represent the sample names and the clustering results of the samples, while the vertical coordinates represent the bacteria and the clustering results of the bacteria. The colors represent the abundance of bacteria in the samples. **(C)** Indicates the RDA analysis for the effects of five biomarkers on the composition of the microbiota community in the guts of *G. cynocephalus*.

*Weissella, Aeromonas, norank_f__norank_o__RsaHf231, ZOR0006*, and *Kocuria* were the most abundant bacterial genera in the guts of *G. cynocephalus* from locations A, B, C, and D during the dry season and from location B during the wet season, respectively. *Achromobacter* was the dominant bacterial genus in the fish guts from locations A and D during the wet season (Figures 7A, B). RDA indicated that *TM7a* had positive effects on the regulation of microbiota community in the fish guts of *G. cynocephalus*, collected during the dry season, while *SM1A02, Rheinheimera, Gemmatimonas*, and *Vogesella* positively regulated those in the wet season (Figure 7C).

**Figure 8:**
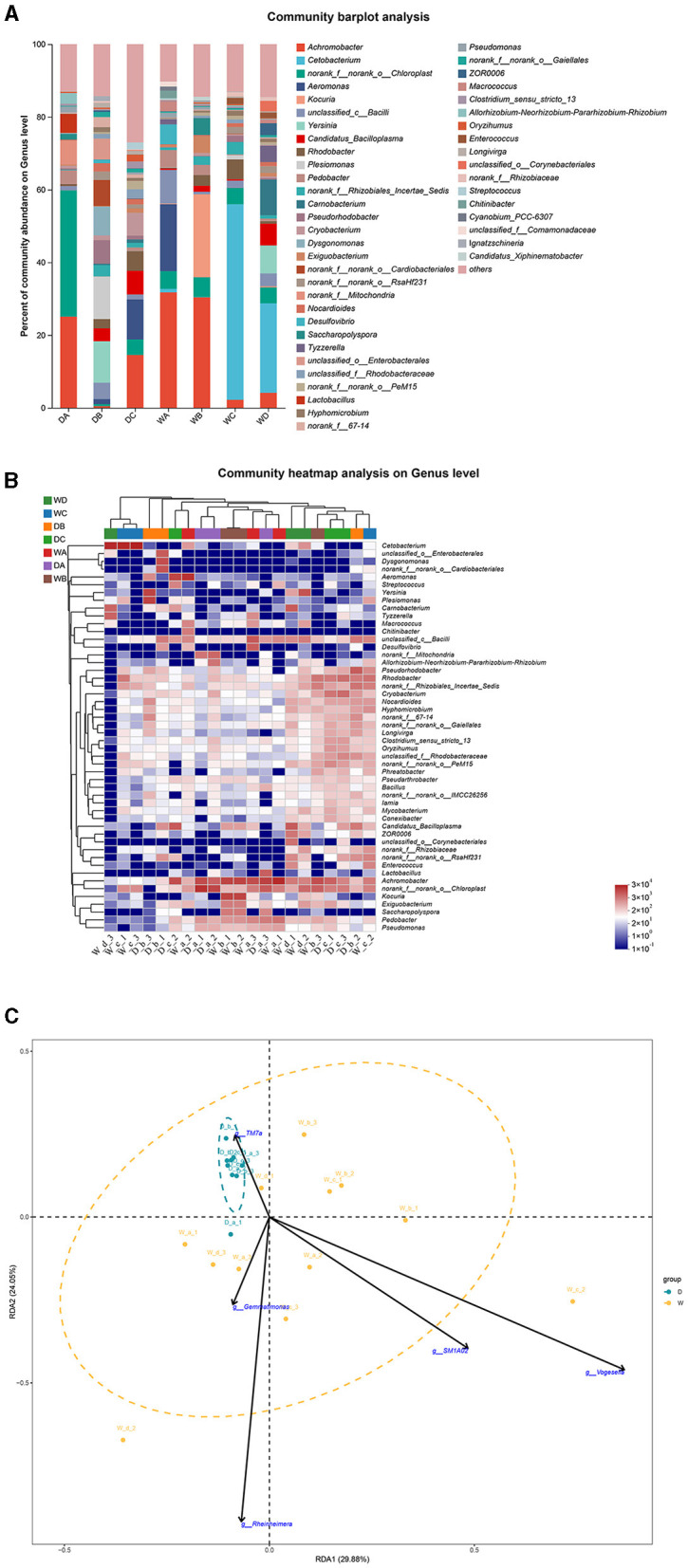
A comparison of microbiota community in the guts of *Leuciscus waleckii* collected from different sample points. D indicates the water samples collected from the dry season. W indicates the water samples collected from the wet season. a, b, c, and d indicate the different locations to collect fish samples. **(A)** Indicates genus distribution as a percentage of the total number of identified sequences in the guts of *L. waleckii* collected from different sample points. **(B)** Indicates the clustering diagram of bacteria at the genus level. The horizontal coordinates represent the sample names and the clustering results of the samples, while the vertical coordinates represent the bacteria and the clustering results of the bacteria. The colors represent the abundance of bacteria in the samples. **(C)** Indicates the RDA analysis for the effects of five biomarkers on the composition of the microbiota community in the guts of *L. waleckii*.

*Norank_f__norank_o__Chloroplast* and *Yersinia* were the most abundant bacterial genera in the guts of *L. waleckii* from locations A and B during the dry season, respectively. *Achromobacter* was the most abundant bacterial genus in the fish guts from location C during the dry season and from locations A and B during the wet season, while *Cetobacterium* was the dominant bacterial genus in the fish guts from locations C and D during the wet season (Figures 8A, B). RDA indicated that *TM7a* positively regulated the formation of microbiota community in the fish guts of *L. waleckii*, collected during the dry season, while *SM1A02, Rheinheimera, Gemmatimonas*, and *Vogesella* had negative effects (Figure 8C).

## 4 Discussion

The Heilongjiang River is one of the largest rivers in the cool temperate zone and has abundant fish resources. However, whether the seasonal changes may result in the changes in microbiota composition in water samples and how the microbiota composition in water samples affects the formation of microbiota diversity in the fish gut are still unclear in the Heilongjiang River. The present study aimed to carry out the spatio-temporal dynamics of microbiota communities in both the water environment and in the fish gut during the dry season and the wet season in the Heilongjiang River in order to analyze the regulatory effects of the water qualities on microbiota composition in water samples and microbiota diversity in fish guts.

In the present study, the pH value and dissolved oxygen during the dry season were generally higher than those of the wet season, while the temperature had opposite regulatory roles, indicating that the water qualities showed the seasonal changes in the Heilongjiang River, which is consistent with the reports in previous studies (Uzukwu et al., 2014; Hee and Suratman, 2016; Achieng et al., 2017).

A number of studies have described seasonal changes in physicochemical parameters as the main factor shaping bacterial and phytoplankton communities (Kršinić et al., 2000; Fuhrman et al., 2006; Gilbert et al., 2006; Kan et al., 2007; Ciglenečki et al., 2015; Pjevac et al., 2015). In the present study, PCA identified two clear separations in the water samples of the wet season and the dry season, indicating that the composition of the microbiota community in the water samples between the dry season and the wet season showed a significant difference. RDA indicated that higher pH values and dissolved oxygen and lower water temperature promoted the formation of microbiota community in the water samples of the dry season and had negative effects on the formation of microbiota community in the water samples of the wet season. A previous study indicated that the abundance of Proteobacteria was significantly affected by the soil pH, toxic metals from pesticides, and terminal electron acceptors, and it can be used as a bacterial indicator for the changes in land use (Kim et al., 2021). Leachate pollution resulted in a 17.73% decrease in Proteobacteria abundance in groundwater, and the phylum Proteobacteria could act as an appropriate indicator for the identification of leachate leakage (Sha et al., 2023). Proteobacteria and Bacteroidota were the most abundant bacterial phyla in the water samples during both the dry season and the wet season. However, the abundance of Proteobacteria in the water samples during the dry season was significantly higher than that of the wet season, while the abundance of Bacteroidota increased in the water samples during the wet season. This is consistent with the previous publication that Bacteroidota thrives under anaerobic conditions, promoting the degradation of macromolecular compounds, such as starch and polysaccharides (Xiao et al., 2023). Five biomarkers with the most difference were identified at the bacterial genus level in the water samples between the dry season and wet season, of which *TM7a* was upregulated in the water samples of the dry season, and *SM1A02, Rheinheimera, Gemmatimonas*, and *Vogesella* were upregulated in the water samples of the wet season. Higher pH values, dissolved oxygen, and lower water temperature positively regulated the formation of *TM7a* and had negative effects on *SM1A02, Rheinheimera, Gemmatimonas*, and *Vogesella*, which was consistent with that of RDA.

The genetic characteristics of microbes in the gut have received significant attention in recent years because the intestinal microbiota is known as the second genome of animals (Meng et al., 2014; Blekhman et al., 2015; Smith C. C. R. et al., 2015; Tzeng et al., 2015; Sauers and Sadd, 2019; Fan et al., 2020; Sanglard et al., 2020). Previous studies have identified that distinct microbiota compositions affected the host homeostasis, physiology, metabolic profile, growth, and vulnerability to disease (Li et al., 2008; Holmes et al., 2012; Nicholson et al., 2012). Some studies have proposed that the fish species is the main influencing factor in shaping the intestinal microbial composition (Li et al., 2012; Liu et al., 2016). Each fish species may shape their own specific microbial symbionts because many factors may influence the shaping of host species-specific ecological traits, including dietary habits, micro-habitat preferences, and mating behavior (Anders et al., 2021). The Proteobacteria, Firmicutes, Bacteroidota, Cyanobacteria, Spirochaetota, Actinobacteria, Campylobacterota, Verrucomicrobiota, and Acidobacteriota became the dominant bacterial phyla in the gut of four omnivorous fishes, habited in the oyster reefs (Bi et al., 2023). The microbiome showed more diversity in the omnivorous cattle than that of herbivorous cattle, and more than 90% of the bacterial sequences were assigned to Firmicutes, Bacteroidetes, Verrucomicrobia, and Proteobacteria at the bacterial phylum level (Lau et al., 2018). Changes in diets also result in a difference in the microbiome community in the intestine of crayfish (Shui et al., 2024), *Sparus aurata* (Piazzon et al., 2022), and *Lates calcarifer* (Huang et al., 2023). The fishes collected in the present study were mainly divided into carnivores (*P. leptocephalus, P. nitidus, G. cynocephalus, S. asotus*), omnivores (*P. glenii, P. lagowskii*, and *L. waleckii*), and herbivores (*X. argentea* and *A. macropterus*). In the present study, Proteobacteria, Firmicutes, Bacteroidota, Cyanobacteria, and Actinobacteria were the dominant bacterial phyla in fish guts, while the relative abundances varied greatly, based on the fish species and dietary habits, indicating dietary habits and fish species may be key factors, affecting the formation and construction of microbiome community in fish gut, collected from the Heilongjiang River.

Environmental factors have significant effects in shaping the establishment of unique microbiota enterotypes (Wu et al., 2011; Akbar et al., 2020; Liu et al., 2021b), revealing a strong bidirectional crosstalk between the host and its microbiome. Water environments were considered to play an important role in the activity and structure of the community in fish (Massana et al., 1997; Eiler and Bertilsson, 2004; Fortin et al., 2004; Kan et al., 2007; Fujii et al., 2012). In the present study, *P. glenii, P. lagowskii, G. cynocephalus*, and *L. waleckii* were the main fish resources, which were collected and identified from at least six sample points. The microbiota community in the same fish species showed a significant difference between different sample points, indicating that the water environment may have regulatory effects on the formation of microbiota community in fish guts from the Heilongjiang River. Further analysis identified that the water environment had regulatory roles in shaping the microbiota community in the guts of *G. cynocephalus* and *L. waleckii* and had limited regulated effects on *P. glenii* and *P. lagowskii*.

## 5 Conclusion

Water qualities showed seasonal changes in the water samples of the Heilongjiang River between the dry season and wet season; of which, pH value, dissolved oxygen, and total dissolved solids were generally higher during the dry season and water temperature was generally higher during the wet season. PCA revealed that the diversity of microbiota varied greatly in the water samples between the dry season and wet season; of which, Proteobacteria was the most abundant bacterial phylum in the water sample of the dry season, and the relative abundance of Bacteroidota was higher in the water sample of the wet season. *TM7a* at the bacterial genus level was significantly upregulated in the water samples of the dry season, while *SM1A02, Rheinheimera, Gemmatimonas*, and *Vogesella* were dramatically upregulated in the water samples of the wet season. Higher pH value, dissolved oxygen, and lower water temperature were identified to positively regulate the formation of *TM7a* and had negative effects on *SM1A02, Rheinheimera, Gemmatimonas*, and *Vogesella*, which was consistent with RDA. Analysis of the composition of the microbiota community in fish guts indicated that dietary habits and fish species may be the two main factors, shaping the microbiota community in fish guts, collected from the Heilongjiang River. Further analysis revealed that the microbiota in the water environment regulated the formation of microbiota community in the guts of *G. cynocephalus* and *L. waleckii* and had a limited regulated effect on *P. glenii* and *P. lagowskii*. The present study identified the spatio-temporal dynamics of microbiota communities in the water environment and in the fish gut during the dry season and the wet season in the Heilongjiang River, providing valuable evidence for the protection of water sources and fish resources in the Heilongjiang River.

## Data availability statement

The original contributions presented in the study are publicly available. This data can be found here: https://www.ncbi.nlm.nih.gov/sra, accession number SRP526333.

## Ethics statement

The animal study was approved by Heilongjiang River Fisheries Research Institute of CAFS Laboratory Animal Welfare and Ethical review. The study was conducted in accordance with the local legislation and institutional requirements.

## Author contributions

HJ: Conceptualization, Formal analysis, Methodology, Validation, Writing – original draft. LL: Conceptualization, Funding acquisition, Project administration, Supervision, Writing – review & editing. WL: Investigation, Methodology, Writing – review & editing. ZZ: Data curation, Formal analysis, Project administration, Writing – review & editing. YX: Data curation, Investigation, Writing – review & editing. DW: Conceptualization, Investigation, Writing – review & editing.
