# Supplementary material for: Identification of the regulatory roles of water qualities on the spatio-temporal dynamics of microbiota communities in the water and fish guts in the Heilongjiang River
Source: Front Microbiol. 2024 Aug 21;15:1435360. doi: 10.3389/fmicb.2024.1435360 (PMC11372393; doi:10.3389/fmicb.2024.1435360)
Supplement: Supplementary file 1 [file Table_1.DOCX]

Supplementary Table 1. Fish samples in wet season

|  | Fish species | Total length (mm) | Body length (mm) | Body weight (g) |
| --- | --- | --- | --- | --- |
| A | *Pelteobagrus nitidus* | 132.77±6.11 | 109.56±5.69 | 17.40±2.43 |
| A | *Parabotia fasciata* | 149.49±6.08 | 93.29±7.11 | 23.43±3.43 |
| A | *Cyprinus carpio* | 273.62±79.04 | 232.36±73.36 | 310.51±21.627 |
| A | *Pseudobagrus ussuriensis* | 113.81±17.21 | 96.27±13.80 | 13.47±5.62 |
| A | *Hemibarbus labeo* | 211.92±17.32 | 173.73±13.28 | 75.35±18.80 |
| A | *Xenocypris argentea* | 135.47±8.14^a^ | 107.36±7.57^a^ | 18.72±3.42^a^ |
| B | *Xenocypris argentea* | 199.53±9.81^b^ | 163.39±8.51^b^ | 64.40±8.61^b^ |
| C | *Xenocypris argentea* | 186.28±6.51^b^ | 162.26±5.12^b^ | 54.07±9.64^b^ |
| A | *Pseudaspius leptocephalus* | 293.04±51.22 | 253.57±49.57 | 200.66±52.98 |
| A | *Sarcocheilichthys nigripinnis* | 117.67±17.56 | 95.48±15.51 | 19.39±9.95 |
| A | *Acheilognathus macropterus* | 87.57±5.86 | 69.08±6.25 | 7.60±1.31 |
| A | *Gobio cynocephalus* | 149.15±19.92^b^ | 124.49±17.35^b^ | 21.85±7.63^b^ |
| B | *Gobio cynocephalus* | 136.86±4.78^b^ | 117.37±2.52^b^ | 20.50±2.34^b^ |
| D | *Gobio cynocephalus* | 115.35±35.55^a^ | 96.24±31.39^a^ | 14.39±14.03^a^ |
| A | *Sarcocheilichthys czerskii* | 108.67±3.79^a^ | 89.23±1.53^a^ | 12.94±1.65^a^ |
| D | *Sarcocheilichthys czerskii* | 124.34±12.69^b^ | 109.93±18.58^b^ | 20.35±5.06^b^ |
| A | *Opsariichthys bidens* | 151.48±15.51^b^ | 124.85±13.51^b^ | 22.50±3.21^b^ |
| B | *Opsariichthys bidens* | 118.72±11.37 | 98.71±11.93 | 11.87±4.01^a^ |
| C | *Opsariichthys bidens* | 129±20.22 | 110.67±21.78 | 20.12±11.14^b^ |
| A | *Leuciscus waleckii* | 188.67±16.74^b^ | 155.48±13.89^b^ | 58.31±15.21^b^ |
| B | *Leuciscus waleckii* | 140.32±15.95^a^ | 114.38±14.73^a^ | 23.23±5.05^a^ |
| C | *Leuciscus waleckii* | 188.43±38.85^b^ | 152.57±33.62^b^ | 53.91±10.78^b^ |
| D | *Leuciscus waleckii* | 191.34±51.11^b^ | 159.28±43.88^b^ | 83.44±28.37^c^ |
| B | *Phoxinus lagowskii* | 98.57±19.66^a^ | 82.94±18.47^a^ | 6.96±2.41^a^ |
| C | *Phoxinus lagowskii* | 91.38±29.09^a^ | 76.54±24.69^a^ | 6.92±1.99^a^ |
| D | *Phoxinus lagowskii* | 127.31±9.16^b^ | 106.48±9.64^b^ | 13.94±4.02^b^ |
| B | *Pseudorasbora parva* | 66.27±11.27^a^ | 56.87±10.21 | 3.12±0.98^a^ |
| C | *Pseudorasbora parva* | 82.43±13.31^b^ | 64.56±13.01 | 5.48±1.67^b^ |
| B | *Rhodeus sericeus* | 69.87±7.23^b^ | 58.44±9.54^b^ | 3.60±1.12^b^ |
| C | *Rhodeus sericeus* | 58.47±5.57^a^ | 46.82±5.03^a^ | 2.20±0.64^a^ |
| D | *Rhodeus sericeus* | 66.17±7.55^b^ | 53.48±5.03^a^ | 3.32±1.09^b^ |
| B | *Perccottus glenii* | 112.34±15.95 | 93.48±12.42 | 19.49±5.35 |
| C | *Perccottus glenii* | 110.52±8.08 | 93.47±7.21 | 18.75±5.69 |
| D | *Perccottus glenii* | 103.48±5.69 | 86.47±4.51 | 14.22±1.76 |
| B | *Silurus asotus* | 269.42±42.1 | 248.75±48.98 | 163.31±82.06^b^ |
| C | *Silurus asotus* | 240.48±13.75 | 210.52±11.59 | 90.53±17.99^a^ |
| C | *Carassius auratus* | 140.49±15.51 | 110.28±11.72 | 48.33±11.11 |
| D | *Phoxinus czekanowskii* | 108.31±11.21 | 90.28±11.59 | 12.33±4.59 |
| D | *Esox reicherti* | 136.28±8.34 | 119.45±7.64 | 12.34±1.52 |
| D | *Misgurnus mohoity* | 110.58±5.19 | 93.46±5.77 | 20.67±1.99 |

Supplementary Table 2. Fish samples in dry season

| Location | Fish species | Total length (mm) | Body length (mm) | Body weight (g) |
| --- | --- | --- | --- | --- |
| A | *Hemibarbus labeo* | 156.48±7.64 | 128.94±4.36 | 28.94±3.83 |
| A | *Hemiculter leucisculus* | 137.48±3.21 | 112.89±2.52 | 14.70±1.13 |
| A | *Acheilognathus macropterus* | 101.89±9.12 | 81.48±9.61 | 11.44±3.62 |
| A | *Sarcocheilichthys nigripinnis* | 156.48±2.89 | 128.47±2.74 | 44.38±2.54 |
| A | *Sarcocheilichthys czerskii* | 108.47±5.29 | 89.97±4.04 | 11.86±1.54 |
| A | *Phoxinus lagowskii* | 97.82±5.86^a^ | 81.37±4.58^a^ | 8.45±1.88^a^ |
| B | *Phoxinus lagowskii* | 121.54±17.04^a^ | 102.38±15.37^a^ | 20.27±5.12^a^ |
| C | *Phoxinus lagowskii* | 101.37±12.37^a^ | 84.92±9.64^a^ | 8.33±2.32^a^ |
| D | *Phoxinus lagowskii* | 149.19±15.39^b^ | 127.47±14.74^b^ | 35.13±9.62^b^ |
| A | *Opsariichthys bidens* | 169.24±24.58 | 141.47±20.82 | 38.32±8.92^a^ |
| C | *Opsariichthys bidens* | 186.13±20.81 | 156.17±20.43 | 69.88±24.86^b^ |
| A | *Pseudaspius leptocephalus* | 183.25±4.04 | 153.67±1.15 | 36.67±3.61 |
| A | *Gobio cynocephalus* | 143.69±13.78^ab^ | 121.06±11.55^ab^ | 24.43±8.44^a^ |
| B | *Gobio cynocephalus* | 123.97±10.02^a^ | 102.88±9.29^a^ | 14.67±3.66^a^ |
| C | *Gobio cynocephalus* | 131.56±15.01^a^ | 113.44±12.53^a^ | 20.05±5.57^a^ |
| D | *Gobio cynocephalus* | 156.28±3.21^b^ | 134.79±2.65^b^ | 38.69±4.29^b^ |
| A | *Xenocypris argentea* | 164.58±27.02 | 135.47±22.50 | 43.86±6.73 |
| A | *Leuciscus waleckii* | 138.47±21.50^a^ | 114.76±19.31^a^ | 21.64±5.68^a^ |
| B | *Leuciscus waleckii* | 135.67±9.01^a^ | 111.69±6.51^a^ | 21.47±3.67^a^ |
| C | *Leuciscus waleckii* | 271.06±12.29^b^ | 228.27±12.89^b^ | 201.54±11.16^b^ |
| B | *Gnathopogon mantschuricus* | 88.45±3.21 | 71.86±1.53 | 5.96±0.41 |
| C | *Gnathopogon mantschuricus* | 84.07±0.58 | 70.32±0.59 | 6.26±0.36 |
| B | *Perccottus glenii* | 101.59±13.58^ab^ | 87.94±7.51^ab^ | 18.17±5.97^b^ |
| C | *Perccottus glenii* | 82.54±17.39^a^ | 67.43±13.43^a^ | 8.15±4.26^a^ |
| D | *Perccottus glenii* | 112.53±19.65^b^ | 97.96±16.82^b^ | 22.75±8.18^b^ |
| B | *Misgurnus mohoity* | 139.28±15.09 | 122.07±14.42 | 10.14±3.52 |
| C | *Misgurnus mohoity* | 138.36±33.29 | 121.57±30.55 | 11.63±5.94 |
| B | *Rhodeus sericeus* | 70.54±5.15 | 56.68±2.08 | 4.15±0.33 |
| B | *Lota lota* | 265.14±50.64 | 247.38±47.62 | 105.21±40.51 |
| B | *Silurus asotus* | 276.61±40.11^a^ | 250.58±38.56^a^ | 123.54±41.54^a^ |
| C | *Silurus asotus* | 315.49±9.49^b^ | 291.83±7.64^b^ | 202.91±47.91^b^ |
| B | *Phoxinus oxycephalus* | 107.18±13.75 | 89.43±11.53 | 9.92±3.68 |
| C | *Phoxinus oxycephalus* | 106.37±8.18 | 89.12±6.08 | 9.01±0.81 |
| C | *Esox reicherti* | 580.94±117.51 | 515.43±99.87 | 1302.86±432.69 |
| C | *Carassius auratus* | 193.66±9.07 | 160.25±6.21 | 129.69±21.52 |
| D | *Phoxinus czekanowskii* | 147.94±19.92 | 124.57±16.77 | 33.49±10.63 |
| D | *Phoxinus phoxinus* | 59.09±1.15 | 50.43±1.02 | 1.98±0.25 |
| D | *Barbatula nudus* | 144.09±12.49 | 123.59±11.93 | 17.20±3.73 |
